# Current Understanding on Antihepatocarcinoma Effects of Xiao Chai Hu Tang and Its Constituents

**DOI:** 10.1155/2013/529458

**Published:** 2013-06-18

**Authors:** Ningning Zheng, Jianye Dai, Huijuan Cao, Shujun Sun, Junwei Fang, Qianhua Li, Shibing Su, Yongyu Zhang, Mingfeng Qiu, Shuang Huang

**Affiliations:** ^1^Center for Traditional Chinese Medicine and Systems Biology, Shanghai University of Traditional Chinese Medicine, Shanghai 201203, China; ^2^Research Center for Traditional Chinese Medicine Complexity System, Shanghai University of Traditional Chinese Medicine, Shanghai 201203, China; ^3^School of Pharmacy, Shanghai Jiao Tong University, Shanghai 200240, China; ^4^Department of Biochemistry and Molecular Biology, Medical College of Georgia, Georgia Health Sciences University, Augusta, GA 30907, USA

## Abstract

Xiao Chai Hu Tang (XCHT), a compound formula originally recorded in an ancient Chinese medical book *Shanghanlun*, has been used to treat chronic liver diseases for a long period of time in China. Although extensive studies have been demonstrated the efficacy of this formula to treat chronic hepatitis, hepatic fibrosis, and hepatocarcinoma, how it works against these diseases still awaits full understanding. Here, we firstly present an overview arranging from the entire formula to mechanism studies of single herb in XCHT and their active components, from a new perspective of “separation study,” and we tried our best to both detailedly and systematically organize the antihepatocarcinoma effects of it, hoping that the review will facilitate the strive on elucidating how XCHT elicits its antihepatocarcinoma role.

## 1. Introduction

The American Cancer Society's estimation for primary liver and bile duct cancers in the United States for 2013 is about 30,640 new cases and 21,670 deaths from these cancers. Liver cancer is even more common in sub-Saharan Africa and Southeast Asia and currently is the most common type of cancers in many countries in these regions [1]. Among various types of liver cancer, hepatocellular carcinoma (HCC) is the most common one, and the majority of them are associated with chronic hepatitis B virus (HBV) or hepatitis C virus (HCV) infections [2, 3]. Chronic viral hepatitis, chemical-induced liver damage, could cause liver fibrosis and cirrhosis, finally leading to liver cancer.

Compound herbal formulas have been used to treat cancers, and many of them have shown the promise to improve the life of cancer patients [4–6]. These compound formulas are usually made of several Chinese medicinal herbs and suppress tumor progression by multiple mechanisms [7]. One of them is called Xiao Chai Hu Tang (XCHT, Sho-saiko-to, in Japanese) that was originally recorded in ancient Chinese medical book *Shanghanlun*. It consists of seven medicinal herbs (*Bupleurum falcatum*,* Scutellaria baicalensis*,* Panax ginseng*,* Zizyphus jujube*,* Pinellia ternate*,* Zingiber officinale*,* and Glycyrrhiza glabra*) and is currently used to treat chronic liver diseases especially chronic hepatitis [8–11]. Data from recent clinical trials convincingly show that XCHT can prevent the development of HCC in patients with cirrhosis, particularly those without HBs antigen [9]. Experimental studies further indicate that XCHT may achieve its effect by reducing hepatocyte necrosis and enhancing liver function. Moreover, XCHT has also been shown to exhibit various anticarcinogenic properties such as induction of apoptosis and suppression of invasion [12, 13].

Chinese herbal medicines are usually used to counteract tumor progression by a formula of multiple herbs rather than a single one. Unfortunately, studies have been mainly focused on defining the mechanism of a single herb or its ingredients. As each herb in XCHT can potentially exert its effect in a distinct mechanism, a formula of seven herbs is expected to reach its full effect by targeting multipathways and multitargets. So we firstly introduce a new perspective of “separation study,” that is, from the entire formula to single herb and their active components, both detailedly and systematically organize the antihepatocarcinoma effects of XCHT. We hope that this review will help XCHT to receive its well-deserved global recognition and to be better appreciated for its clinical use to treat liver cancers.

## 2. Xiao Chai Hu Tang (XCHT)

### 2.1. Clinical Trials of XCHT

XCHT has long been used in clinical trials for the study and treatment of liver diseases. Some XCHT clinical trials (including the clinical trials mentioned above and some other trials [14–17] conducted in China) are summarized in Table 1. 

### 2.2. Experimental Studies of XCHT

The potential tumor-suppressing effect of XCHT was reported in 1994, in which XCHT was found capable of inhibiting the proliferation of KIM-1, a human hepatocellular carcinoma cell line and KMC-1, a cholangiocarcinoma cell line [18]. Later on, XCHT was shown to diminish not only the growth of various cancer cell lines but also in vivo tumor outgrowth in xenograft model [19–21].

In addition to its tumor-suppressing role, Chang et al. studied the effect of XCHT on HBV replication in HepG2 2.2.15 cell model [22]. Their study showed that XCHT reduced HBV production and HBeAg expression without altering the level of HBsAg. Although XCHT can also block Coxsackie B type 1 virus infection in CCFS-1 cells through the induction of Type I interferon expression [23], the mechanism responsible for XCHT-mediated suppression of HBV production awaits being defined. 

Hepatic fibrosis and liver cirrhosis result from wound healing of ongoing hepatocellular damage caused by chronic liver injuries [13]. Most of HCCs ensue in a cirrhotic liver [24]. Kusunose et al. created an animal model that reflected various stage-liver injuries and used this model to determine under what condition XCHT extract could improve hepatic inflammation and fibrosis [25]. Their study found that the ability of XCHT extract was limited to a certain degree which was expressed by levels of respective parameters (AST, ALT, TGF-β, hydroxyproline, and the ratio of liver fibrosis area). Chen et al. later elucidated the mechanism pertinent to XCHT's antifibrosis capability by assessing its effect on the expression of those growth factors and cytokines important for the activation of hepatic stellate cells (HSCs) [26]. They showed that XCHT downregulated the levels of stellate cell activation-essential TGFβ1, platelet derived growth factor (PDGF), and IL-1β while stimulated the production of stellate cell activation-inhibitory TNF*α*.

## 3. Individual Herbs and Active Components in XCHT

In TCM, XCHT is a classical formula to treat the typhoid lesser yang syndrome. The compound prescription has its formulating principle. Among the seven herbs included in XCHT, *Bupleurum falcatum *is the “monarch” and *Scutellaria baicalensis *is the “minister,” which are the principal herbs in this formula. *Panax ginseng*, *Zizyphus jujube*, *Pinellia ternate*, and* Zingiber officinale *are the “assistant” while *Glycyrrhiza glabra *is the “guide” [27]. *Bupleurum* has the effect of upraising and dispersing the pathogen and soothing the meridian Qi. *Scutellaria* has the effect of clearing and down-sending heat with bitter-cold, as well as eliminating the heat. *Panax ginseng *and* Zizyphus jujube *work compatibly to tonify Qi and fortify the spleen, thus strengthening the body and eliminate pathogens; *Pinellia ternate *and* Zingiber officinale *work together to regulate the stomach Qi and prevent vomit; and the “guide” *Glycyrrhiza glabra* is used to harmonize the other herbs. Nowadays, XCHT is used to treat common cold, chronic hepatitis, hepatic cirrhosis, bile reflux gastritis, cholecystitis, acute pancreatitis, and so forth, which belong to lesser yang syndrome.

### 3.1. *Bupleurum falcatum*

The root of *Bupleurum falcatum* L. (Umbelliferae), especially *B. chinense* from mainland China and *B. falcatum* from Japan [28, 29], is also called Bupleuri Radix ((BR) Chaihu, in Chinese and Saiko in Japanese) and is one of the principal herbs in XCHT. Early study examined the hepatoprotective effect of several BR extracts with dimethylnitrosamine- (DMN-) induced hepatic fibrosis rat model and these extracts appeared to prevent fibrosis by improving liver function and modulating the levels of relevant cytokines [29]. Recent studies also suggest BR extracts as potent antioxidant agents because they are able to decrease L-thyroxine-induced hypothyroidism and to enhance the liver antioxidant defense systems [30]. 

**Figure 1:**
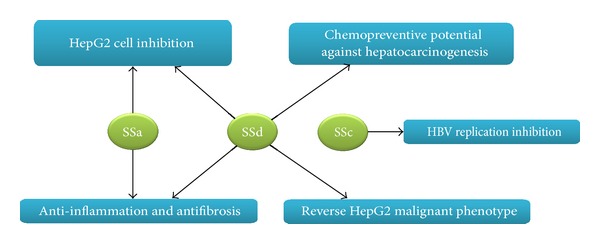
Antitumorigenesis effect of active components in *Bupleuri* radix.

Some saikosaponins, which are the active ingredients of BR, have been found to suppress hepatic fibrosis [31, 32], hepatocarcinoma [33–36], and HBV infection [37] and improve chemotherapy [38]. The different mechanisms through which saikosaponins suppressed hepatocarcinoma were summarized in Figure 1. Saikosaponin a (SSa) was shown to effectively inhibit CCl_4_-induced liver inflammation and fibrosis in SD rats by simultaneously blocking the production of hepatic proinflammatory cytokines/growth factors (TGFβ1 and hydroxyproline) and increasing the expression of anti-inflammatory cytokine IL-10 [31]. Saikosaponin d (SSd) was found to suppress hepatic fibrosis through the downregulation of TNF-*α*, IL-6, and NF-*κ*B activities [32]. SSa may inhibit HepG2 growth by increasing the levels of p-15INK4a and p-16INK4b (cyclin-dependent kinase inhibitors) in a protein kinase C (PKC) [33] and/or extracellular signal-regulated kinase (ERK) signaling pathway-dependent manner [34]. SSd markedly reduced the liver nodule, tumor cell invasion while increased cellular atypia in xenograft model [35]. It appeared that SSd exerted its action by diminishing the expression of highly expressed cyclooxygenase 2 (COX-2) and CCAAT/enhancer-binding protein β (C/EBPβ) in tumor cells and macrophages of liver tumors [35]. In a study reported by Zhu et al. [36], SSd was shown capable of reversing the malignant phenotype of HepG2 cells. SSd-treated HepG2 cells grew and migrated at slower rate, had decreased volume ratios of nucleus to plasma and small round cell shape. At molecular level, SSd decreased the level of alpha-fetoprotein (AFP) and enhanced the expression of cell cycle inhibitor p27. Moreover, Chiang et al. showed that HBV-containing human hepatoma cells (2.2.15 cells) treated with saikosaponin c (SSc) secreted significantly less HBeAg into culture medium and had reduced HBV DNA replication [37]. Although not directly using liver cancer cells, SSa and SSd were also shown to sensitize cervical (HeLa and Siha), ovarian (SKOV3), and lung cancer cells (A549) to cisplatin-induced cell death by inducing the production of reactive oxygen species (ROS) and activation of caspases [38].

### 3.2. *Scutellaria baicalensis*

The dry root of *Scutellaria baicalensis*, Scutellaria radix ((SR) Hangqin, in Chinese ) is another principal herb in XCHT. Accumulating evidences indicate that wogonin, baicalein, and baicalin are the principal active components in SR [39]. SR has been widely used to treat hyperlipemia, atherosclerosis, and hypertension. Recent studies with various model systems suggest that SR also possesses a potent cytostatic [40–42], anti-inflammatory [43] and antiviral capabilities [44, 45]. 

Antitumorigenesis ability of SR was reported by Gao et al. in human lung cancer cells (SK-MES-1, SK-LU-1, and A549) [42]. Their study showed that the absolute ethanol extracts of *Scutellaria baicalensis*, baicalin, baicalein, and wogonin all displayed a concentration- and time-dependent cytotoxicity to lung cancer cells while were only weakly cytotoxic to the normal human lung fibroblasts. Jung et al. later discovered that *Scutellaria baicalensis* is an anti-inflammatory agent because it decreases histamine release and inhibits the passive cutaneous anaphylaxis reaction in SD rats [43]. Antiviral effect of *Scutellaria baicalensis* was shown by Tang et al., in which it was shown to significantly inhibit the replication of HCV RNA in HCV-infected nude mice [44]. Later study revealed that the aqueous extract of *Scutellaria baicalensis* was also able to suppress the replication of lamivudine-resistant HBV mutant in human hepatoma cells by suppressing HBV core promoter activity [45].

**Figure 2:**
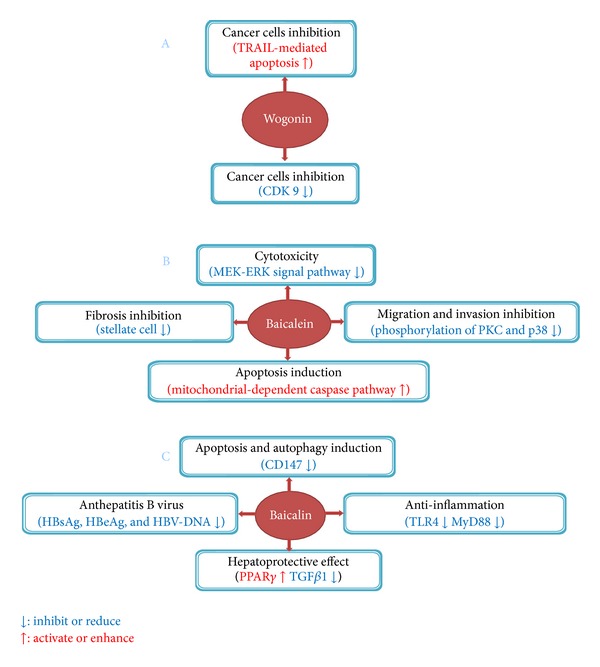
Suppressive effect of active components of *Scutellaria* radix on liver tumorigenesis and fibrosis.

Besides the studies performed with *Scutellaria baicalensis*, active components of it have also been extensively investigated (Figure 2). TNF-related apoptosis-inducing ligand (TRAIL) has been recognized as a promising anticancer agent because it kills tumor cells without damaging normal tissues [46, 47]. However, resistance to TRAIL is frequently seen in various tumor types. Ding et al. found that wogonin and structurally related natural flavones apigenin and chrysin overcame TRAIL resistance by downregulating the level of c-FLIP (a key inhibitor of death receptor signaling) and up-regulating TRAIL receptor 2 (TRAIL-R2) expression in human T-cell leukemia virus type 1- (HTLV-1-) associated adult T leukemia/lymphoma (ATL) cells [48]. They further showed that these flavones could enhance TRAIL-mediated apoptosis in a wide variety of cancer cell types including hepatocellular carcinomas (HepG2), breast (MDA-MB-231), colon (HT-29), and pancreatic cancer cells (Capan-1) as well as melanoma cells (SK-MEL-37) [48], implicating the use of flavones as an adjuvant for TRAIL-mediated anticancer therapy. In another study, Polier et al. initially showed that wogonin and flavones are inhibitors of cyclin-dependent kinase 9 (CDK9) and can effectively block phosphorylation of the carboxy-terminal domain of RNA polymerase II at Ser2, which in turn reduces RNA synthesis and subsequent downregulation of antiapoptotic protein myeloid cell leukemia 1 (Mcl-1), leading to significant apoptosis in a variety of human cancer cells [49]. However, wogonin-induced apoptosis of human hepatocarcinoma cells was found to be accompanied with Bax increase and Bc1-2 decrease [50, 51]. Anti-HBV effect of wogonin was also found in vitro and in vivo [52], with the HBV antigen and HBV DNA level reduction.

Baicalein, a flavonoid extracted from SR, has been shown to possess potent antitumorigenesis capability toward liver cancer cells. For example, baicalein is highly cytotoxic to HCC cell lines and exerts its cytotoxicity by reducing mitochondrial transmembrane potential and subsequent cytochrome c release and caspase-3/9 activation. Disruption of MEK-ERK signaling pathway is at least partially responsible for baicalein-induced cytotoxicity [53, 54]. When used in vivo, baicalein can significantly inhibit tumor growth of HCC xenografts [53]. In another study, baicalein was reported to block cell migration and invasion of human hepatoma cells through multiple mechanisms including the suppression of MMP-2, MMP-9, and uPA expressions, blockage of NF-*κ*B activation, and decreasing the phosphorylation levels of PKC*α* and p38 MAPK activities [55]. In a recent study, Sun et al. showed that baicalein dose dependently decreased AST, ALT, hyaluronic acid, laminin, and procollagen type III (PCIII) in serum as well as hydroxyproline and MMPs in liver in CCl_4_-induced liver fibrosis model [56]. Moreover, baicalein also alleviated inflammation, destruction of liver architecture, collagen accumulation and expression of PDGFβ receptor, thus preventing the activation of stellate cells and liver fibrosis [56].

Baicalin is also an important active component included in SR. Zhang et al. [57] found that baicalin induced apoptosis with downregulation of glycosylated immunoglobulin superfamily transmembrane protein CD147 expression in SMMC-7721 cells, and interestingly, this effect was accompanied with cell autophagy. This study firstly suggested that baicalin induced autophagy cell death in SMMC-7721 cells and revealed a new mechanism for the anticancer effects of baicalin. Qiao et al. [58] studied the antihepatic fibrosis effect of baicalin and found that transplantation with baicalin-treated mesenchymal stem cells in combination with baicalin administration had the best therapeutic effect for hepatic fibrosis. This may further introduce a new therapeutic regimen for some liver diseases. Baicalin combined with oxymatrine showed better effect against HBV replication than oxymatrine in vitro, which was proved by Cheng et al. [59]. In aspects of the potential protective effect on liver injury, baicalin was also researched in many experiments [60–62]. Activation of peroxisome proliferator-activated receptor (PPAR*γ*) signaling pathway, and Toll-like receptor 4- (TLR4-) mediated inflammatory responses were involved in the protective effect.

### 3.3. *Panax ginseng*

Ginseng products are regularly consumed worldwide for the purpose of increasing vitality [63]. Recently, many studies have shown the chemopreventive or adjuvant effect of it [64]. A study involving two cases of control (905 pairs and 1987 pairs, resp.) and a cohort (4675 subjects) demonstrated the benefit of ginseng use for cancer prevention as ginseng use was found to be nonorgan-specific cancer preventive, and its effect depends on the frequency of ginseng intake [65].

In addition to ginseng's preventive effect toward cancer, evidences from experimental studies also suggest its direct role to suppress liver tumorigenesis. Wu et al. showed that ginseng lowered the rate of hepatoma development and prolonged life span on diethylnitrosamine (DEN) rat liver cancer model [66]. Kwon et al. found that oral administration of ginseng decreased the levels of AST and ALT, number of degenerative cells, and area of connective tissue in the liver of dogs during liver regeneration after partial hepatectomy [67]. Bak et al. showed that the use of ginsengs' essential oil diminished the production of ROS and restored both the activities and expression of antioxidant enzymes including superoxide dismutase (SOD), glutathione peroxidase (GPx) and catalase (CAT) in H_2_O_2_-treated HepG2 cells or CCl_4_-treated mice [68]. The effect of ginseng appears to be mediated by a simultaneous inhibition of JNK, ERK, and p38 activities and upregulated expression of antioxidant enzyme expression in the liver.

**Figure 3:**
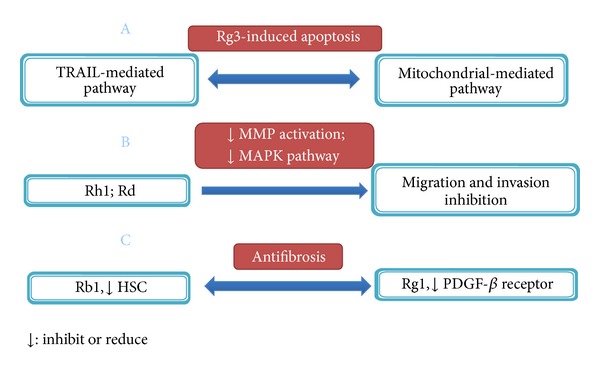
Antitumorigenesis effect of active components in ginseng.

Components of ginseng have also been investigated for their inhibitory effect on liver tumorigenesis (Figure 3). Lee et al. showed that 20(S)-ginsenoside Rg3, a steroidal saponin was able to sensitize HCCs, but not normal hepatocytes to TRAIL-induced cell death [69]. Importantly, Rg3 was found to be well tolerated in animals and significantly enhance the therapeutic efficacy of TRAIL in xenograft models [69]. And other studies [70, 71] suggested that intrinsic apoptotic pathway may be involved in the inhibitory effect of Rg3 on hepatocellular carcinoma cell lines. To elucidate the mechanism associated with ginseng extract-induced cell death, Park et al. showed that primary ginsenosides Rg3 and Rh2 are mainly responsible for ginseng's effect and they act by directly activating mitochondrial-dependent apoptotic pathway and inducing the production of intracellular ROS [72]. 

Components of ginseng also exhibit their tumor-suppressing capability by blocking cell migration and invasion. Yoon et al. found that ginsenoside Rh1 inhibited HepG2 cell migration and invasion by abrogating MAPK-dependent MMP-1 expression [73]. Similar effect was also observed with ginsenoside Rd in HepG2 cells [74]. As excess production of extracellular matrix by activated hepatic stellate cells (HSCs) is the major cause of liver fibrosis, Lo et al. determined the potential protective effect of ginseng components toward liver fibrosis. They revealed that ginsenoside Rb1 exerted an antifibrotic effect under H_2_O_2_ oxidative stress by inhibiting HSC activation/proliferation [75]. Another ginsenoside, Rg1, has also been shown to prevent thioacetamide-induced hepatic fibrosis in rats by intercepting NF-*κ*B-mediated PDGFβ receptor expression [76].

### 3.4. Other Herbs in XCHT

Besides the three herbs that have been described above, the remaining herbs in XCHT are *Zizyphus jujube*, *Pinellia ternate*,* Zingiber officinale*,* and Glycyrrhiza glabra* and act as adjuvant herbs in this compound formula. *Zizyphus jujube* in XCHT is prescribed by Traditional Chinese Medicine doctors to calm mind based on its ability to invigorate the spleen and nourish the blood. Recent experimental evidences showed that it was able to attenuate chemical-induced liver injury in rats [77, 78]. *Pinellia ternate* is another herb in XCHT while the mechanism study about its antihepatocarcinoma effect is rare. Although it appears to boost the efficacy of XCHT, how it does this awaits being further explored. *Zingiber officinale*, a species used for over thousand years, appears to display anticancer, anti-inflammatory, and chemopreventive effects in both in vitro and in vivo models [79, 80]. 6-shogaol and 6-gingerol are the two active compounds of ginger, and their effects of apoptosis induction [81], hepatocarcinoma invasion inhibition [82] and anti-hepatoxicity [83] were also studied. Licorice is the dried root of *Glycyrrhiza uralensis* Fisch, and both the extract [84, 85] and its active component glycyrrhizin [86, 87] were explored for their hepatoprotective capability. Though these adjuvant herbs are also essential in XCHT from the view point of TCM, modern mechanism studies about their anti-hepatocarcinoma effects are relatively less than *Bupleurum falcatum*, *Scutellaria baicalensis,* and *Panax ginseng*. So we did not summarize the antihepatocarcinoma-related effects of these adjuvant herbs as detailedly as the former three herbs here.

## 4. Summary and Prospect

**Figure 4:**
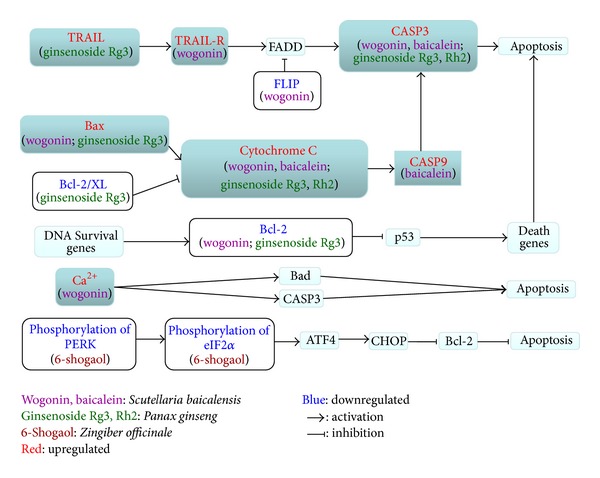
Tumor cell apoptosis-related pathways targeted by active components.

**Figure 5:**
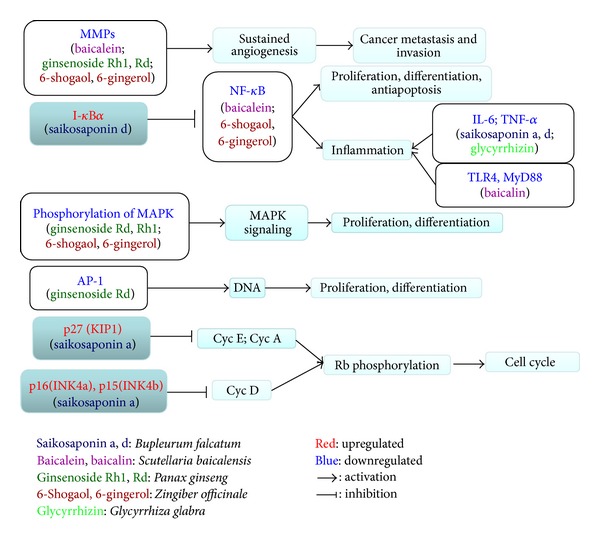
Tumor- and fibrosis-related pathways targeted by active components.

**Figure 6:**
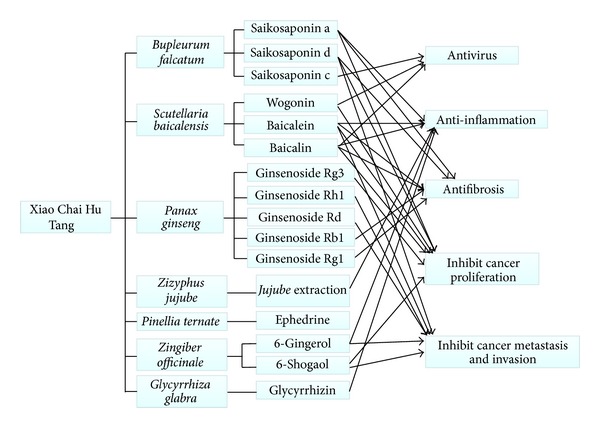
Antihepatocarcinoma effects of XCHT.

Besides the summary above, experimental studies of the active components in the herbs on antihepatocarcinoma-related effects are further summarized in Tables 2, 3, 4, and 5 based on their distinct cellular aspects, and some other carcinoma cell lines were also included in Tables 2–5 to better elucidate the mechanism. What is more, to systematically organize the mechanism, we searched Kyoto Encyclopedia of Genes and Genomes (KEGG) database (http://www.genome.jp/kegg/) to connect the factors and pathways together which were targeted by the active components, as presented in Figures 4 and 5. Though the two figures cannot present all the targets summarized in our paper, they still could illustrate the mechanism from a more systematical aspect. On the basis of Figures 1 to 5, we depicted Figure 6 to better display the antihepatocarcinoma effects at three different levels of formula, herbs, and components. From them, it is apparent that components of XCHT possess a broad spectrum of activities ranging from antitumor, anti-inflammation to fibrosis-protective effects. Some of the components directly target on tumor growth, metastasis, and invasion, while others act on inflammation and fibrosis related pathways or antiviral process to prevent further virus-facilitated tumorigenesis. Particularly, many of these components share analogous factors and pathways. 

Besides tumor growth, metastasis and invasion, angiogenesis is also an essential pathological component of cancer. Antiangiogenic therapy is considered to limit tumor progression [88]. Research about the anti-angiogenic effect on hepatocarcinoma of XCHT has not been seen, while some reseachers have studied the effect of herbs and active compounds in it on the angiogenic action [89–91]. Interestingly, different compounds in ginseng (ginsenoside Rg3 and Rg1) possessed contrary angiogenic action (antiangiogenic and angiogenic effects) [89, 90]. What is more, as we summarized in our review, components of XCHT may affect various biochemical pathways, many of which are related to angiogenesis [92]. So it is also worthy to deeply study XCHT's effect on the angiogenic action for tumor treatment.

Our overview is based on a perspective of “separation study,” that is, arranging from the entire formula to each herb and their active components. This method may comprehensively utilize and deeply excavate the existing researches. It is difficult to directly elucidate a complex formula, while it will be easier when we separately study the constituents in it. Currently, to unequivocally interpret the antihepatocarcinoma effect of XCHT and the active ingredients contained in it is still difficult. The underlying reasons are severalfold. The first is that the knowledge on how each component works is still not sufficient and their respective targets are still needed to be identified. The second is how these components work in concert to achieve therapeutic effect is not understood. As the philosophy of Traditional Chinese Medicine, compound herbal formulas are used to treat disease by regulating human body globally, targeting multiple pathways and targets. This characteristic may be better coincident with cancer which may be induced by multiple factors. Tumor growth, metastasis, and invasion are the final features that we notice, but the underlying mechanism may be related with many factors. The method of “separation study” should be combined with bioinformatics, meaning that we may use bioinformatics to integrate these separated studies. So more innovative researches and novel strategies will have to be employed to fully understand the mechanisms responsible for XCHT's therapeutic effects.

## Figures and Tables

**Table 1:** Some clinical trials of XCHT.

Author; year	Cases	Research design	Results
Hirayama et al. [8]; 1989	222 chronic hepatitis subjects	Double-blind, multicenter	The difference of the mean value of AST and ALT between the XCHT group and placebo group was significant; a tendency towards a decrease of HBeAg and an increase of anti-HBe antibodies was also observed in patients with chronic active type B hepatitis
Oka et al. [9]; 1995	260 cirrhotic subjects	Randomized, controlled	The cumulative incidence curve for 5 years of the trial group (XCHT combined with conventional drugs) was lower while the survival curve for 5 years of the trial group was higher compared with control group (conventional drugs). The difference was significant for patients without HBs antigen
Deng et al. [11]; 2011	24 chronic hepatitis C subjects	A single arm phase II study	Improvement of AST (16 subjects) and ALT (18 subjects) was observed; 9 subjects showed improvement in histology activity index scores
Bo and Du [14]; 2006	96 chronic hepatitis B subjects	Randomized, controlled	Experiment group (XCHT combined with *α*-interferon) showed better effect in aspects of ALT improvement and HBeAg negative transform than *α*-interferon treatment group
Li et al. [15]; 2001	110 chronic hepatitis B subjects	Randomized, controlled	ALT, total bilirubin, and serum liver fibrosis indexes were decreased in combination treatment group (XCHT and *γ*-interferon) and the difference was significant compared with *γ*-interferon treatment group
Sun et al. [16]; 2003	94 chronic hepatitis B with fibrosis subjects	Randomized, controlled	The liver function was improved and serum liver fibrosis indexes were decreased; the difference was significant between combination treatment group (XCHT and oxymatrine) and controlled group (reduced glutathione and vitamin treatment)
Wu [17]; 2009	142 chronic hepatitis B with cirrhosis subjects	Randomized, controlled	The liver function was improved and serum liver fibrosis indexes were decreased; the difference was significant between XCHT treatment group and controlled group (hepatic protective drug and antifibrosis drug treatment)

**Table 2:** Apoptosis-inducing effects of active components.

Author; year	Animal or cell	Active components	Factors and pathways
Wu and Hsu [33]; 2001	HepG2 cells	Saikosaponin a	PKC signaling pathway involved; CDK inhibitor p-15^INK4a^ and p-16^INK4b ^mRNA and protein↑
Wu [34]; 2003	HepG2 cells	Saikosaponin a	ERK signaling pathway involved; CDK inhibitor p-15^INK4a^ and p-16^INK4b^ mRNA and protein↑
Wang et al. [38]; 2010	Cervical cancer (HeLa and Siha); ovarian cancer (SKOV3); non-small-cell lung cancer (A549) cell lines	Saikosaponin a, d	ROS↑; caspase pathway activation
Ding et al. [48]; 2012	HTLV-1-associated ATL	Wogonin	c-FLIP↓; TRAIL-R2 expression↑
Polier et al. [49]; 2011	The human colorectal carcinoma (HCT116); the human leukemic T-cell line (CEM); the adult T-cell leukemic cell line (SP)	Wogonin	CDK9↓; antiapoptotic protein Mcl-1↓
Wang et al. [50]; 2006	Human hepatoma cell line (SMMC-7721)	Wogonin	Bax↑Bc1-2↓
Lin et al. [51]; 2011	Human osteosarcoma cell line (U-2 OS)	Wogonin	ROS↑ intracellular Ca2^+^↑ caspase-3 activity↑; Bad, Bax, cytochrome c↑; mitochondrial membrane potential↓
Liang et al. [53]; 2012	HCC cell lines; mice with HCC xenografts	Baicalein	Mitochondrial transmembrane potential↓; caspase-9, caspase-3↑; phosphorylation of MEK1, ERK1/2, and Bad↓
Kuo et al. [54]; 2009	Human hepatoma J5 cells	Baicalein	Mitochondrial-dependent caspase activation pathway involved (mitochondrial cytochrome c release; activation of caspase-9 and -3; the ratio of Bax/Bcl-2↑)
Zhang et al. [57]; 2012	HCC cell line (SMMC-7721)	Baicalin	CD147↓ cell apoptosis and autophagy were induced
Lee et al. [69]; 2012	HepG2, SK-Hep1, Huh-7, and Hep3B cell lines; mouse xenograft model	Ginsenoside Rg3	Promoting TRAIL-induced apoptosis
Zhang et al. [70]; 2012	Human hepatocellular carcinoma cell lines (SMMC-7721; HepG2)	Ginsenoside Rg3	Gene expression of caspase-3; Bax↑; Bcl-2↓
Jiang et al. [71]; 2011	Hep1-6 and HepG2 cells; liver tumor-bearing C57Bl6 mice	Ginsenoside Rg3	Mitochondrial pathway involved (mitochondria membrane potential↓; caspase-3 activation↑; Bax↑ Bcl-2 and Bcl-XL↓)
Park et al. [72]; 2012	Human hepatocellular carcinoma cells (Hep3B)	Ginsenoside Rg3, Rh2	Activating the mitochondrial pathway (ROS↑; Bax↑ Bcl-2↓; cytochrome c↑; activation of caspase-3)
Hu et al. [81]; 2012	SMMC-7721, BEL-7404, HL-7702 cells; SMMC-7721 xenograft-bearing mouse	6-Shogaol	Unfolded protein response (UPR)↑; PERK/eIF2*α* pathway↑; eIF2*α* phosphorylation↓; caspase 3↑

**Table 3:** Metastasis and invasion-inhibitory effects of active components.

Author; year	Animal or cell	Active components	Factors and pathways
Zhu et al. [36]; 2011	HepG2 cells	Saikosaponin d	Cell growth↓ cell migration↓; AFP↓; p27 mRNA expression↑
Chiu et al. [55]; 2011	human hepatoma cell lines (HA22T/VGH and SK-Hep1)	Baicalein	The gelatinolytic activities of MMP-2, MMP-9, uPA↓; NF-*κ*B activation↓; phosphorylation of PKC*α* and p38 proteins↓
Yoon et al. [73]; 2012	HepG2 cells	Ginsenoside Rh1	Inactivation of MAPKs; MMP-1 gene expression↓
Yoon et al. [74]; 2012	HepG2 cells	Ginsenoside Rd	MAPK signaling↓; activation of AP-1↓; expression of MMP-1, MMP-2, and MMP-7↓
Weng et al. [82]; 2012	Hep3B cells	6-Shogaol; 6-gingerol	MMP-2 and MMP-9↓; uPA↓; the phosphorylation of MAPK↓; PI3K/Akt signaling↓; NF-*κ*B activation↓

**Table 4:** Inflammation and fibrosis inhibitory effects of active components.

Author; year	Animal or cell	Active components	Factors and pathways
Wu et al. [31]; 2010	CCl_4_-induced liver inflammation and fibrosis rats	Saikosaponin a	Proinflammatory cytokines TNF-*α*, IL-1*β*, IL-6↓; anti-inflammatory cytokine IL-10↑; TGF-*β*1 and hydroxyproline↓; NF-*κ*B↓
Dang et al. [32]; 2007	Liver fibrotic rats	Saikosaponin d	TNF-*α*, IL-6 and NF-*κ*Bp65 expression↓; I-*κ*B*α* activity↑
Sun et al. [56]; 2010	CCl_4_-induced liver fibrosis rats	Baicalein	AST, ALT, hyaluronic acid, laminin, and PDGF-β receptor↓; hydroxyproline, MMPs↓
Qiao et al. [60]; 2011	CCl_4_-induced liver injury rats	Baicalin	PPAR*γ*↑; TGFβ1↓
Kim and Lee [62]; 2012	Ischemia/reperfusion injured rats with alcoholic fatty liver	Baicalin	Toll-like receptor 4 (TLR4)↓; myeloid differentiation primary response protein My88↓
Lo et al. [75]; 2011	HSCs	Ginsenoside Rb1	HSCs activation and proliferation↓; expression of collagen, TGF-*β*1, MMP-2, and TIMP-1↓
Geng et al. [76]; 2010	Thioacetamide-treated rats; HSCs	Ginsenoside Rg1	AST, ALT, hydroxyproline↓; HSCs↓; PDGF-*β* receptor expression↓
Sabina et al. [83]; 2011	Acetaminophen-treated mice	6-gingerol	The hepatic marker enzymes (AST, ALT, and ALP) and total bilirubin in serum↓; hepatic malondialdehyde formation↓; liver antioxidant status↑
Gumpricht et al. [86]; 2005	Rat hepatocytes exposed to GCDC	Glycyrrhizin; 18-beta-glycyrrhetinic acid	Glycyrrhizin-enhanced GCDC induced cell apoptosis; 18-beta-glycyrrhetinic acid reduced cell necrosis and protected against GCDC-induced cell apoptosis
Lee et al. [87]; 2007	CCl_4_-induced liver injury rats	Glycyrrhizin	Liver function improvement; proinflammatory mediators (TNF-*α*, inducible nitric oxide synthase, and COX-2)↓; heme oxygenase-1↑;

**Table 5:** Antiviral effect of active components.

Author; year	Animal or cell	Active components	Factors and pathways
Chiang et al. [37]; 2003	HBV-transfected human hepatoma cells	Saikosaponin c	HBeAg↓; HBV DNA↓
Guo et al. [52]; 2007	HepG2.2.15; HBV-infected ducks; HBV-transgenic mice	Wogonin	HBsAg and HBeAg↓; HBV DNA↓
Cheng et al. [59]; 2006	HepG2.2.15 cells	Baicalin	HBsAg and HBeAg↓; HBV DNA↓

## Abbreviations

ATL: Adult T-cell leukemia/lymphoma
ALP: Alkaline phosphatase
AFP: Alpha-fetoprotein
AP-1: Activator protein-1
ATF4: Cyclic AMP-dependent transcription factor
c-FLIP: Cellular FLICE inhibitory protein (CASP8 and FADD-like apoptosis regulator)
CASP: Caspase
CycD: Cyclin D
CDK9: Cyclin-dependent kinase 9
COX-2: Cyclooxygenase 2
C/EBPβ: CCAAT/enhancer-binding protein β
CD147: Glycosylated immunoglobulin superfamily transmembrane protein
CHOP: C/EBP homologous protein
DEN: Diethylnitrosamine
ERK: Extracellular signal-regulated kinase
eIF2*α*: Translation initiation factor
FADD: FAS-associated death domain protein
GCDC: Glycochenodeoxycholic acid
HTLV-1: Human T-cell leukemia virus type1
HSCs: Hepatic stellate cells
HBsAg: Hepatitis B surface antigen
HBeAg: Hepatitis B e antigen
I-*κ*B*α*: NF-kappa-B inhibitor alpha
Mcl-1: Myeloid cell leukemia-1
MMP: Matrix metalloproteinase
MAPK: Mitogen-activated protein kinase
My88: Myeloid differentiation primary response protein
PKC: Protein kinase C
PDGF: Platelet derived growth factor
PERK: PKR-like endoplasmic reticulum associated kinase (eukaryotic translation initiation factor kinase)
PPAR*γ*: Peroxisome proliferator-activated receptor *γ*
ROS: Reactive oxygen species
Rb: Retinoblastoma-associated protein
TRAIL-R2: TNF-related apoptosis-inducing ligand receptor 2
TIMP: Tissue inhibitor of metalloproteinase
TGFβ1: Tumor growth factor β1
TLR4: Toll-like receptor 4
uPA: Urokinase plasminogen activator.

## References

[1] http://www.cancer.org/cancer/livercancer/overviewguide/liver-cancer-overview-key-statistics.

[2] El-Serag HB (2012). Epidemiology of viral hepatitis and hepatocellular carcinoma. *Gastroenterology*.

[3] Akriviadis EA, Llovet JM, Efremidis SC (1998). Hepatocellular carcinoma. *British Journal of Surgery*.

[4] Hyodo I, Amano N, Eguchi K (2005). Nationwide survey on complementary and alternative medicine in cancer patients in Japan. *Journal of Clinical Oncology*.

[5] Li XQ, Ling CQ (2012). Chinese herbal medicine for side effects of transarterial chemoembolization in liver cancer patients: a systematic review and meta-analysis. *Zhong Xi Yi Jie He Xue Bao*.

[6] McQuade JL, Meng Z, Chen Z (2012). Utilization of and attitudes towards traditional Chinese medicine therapies in a Chinese cancer hospital: a survey of patients and physicians. *Evidence-Based Complementary and Alternative Medicine*.

[7] Ruan W-J, Lai M-D, Zhou J-G (2006). Anticancer effects of Chinese herbal medicine, science or myth?. *Journal of Zhejiang University. Science. B*.

[8] Hirayama C, Okumura M, Tanikawa K, Yano M, Mizuta M, Ogawa N (1989). A multicenter randomized controlled clinical trial of Shosaiko-to in chronic active hepatitis. *Gastroenterologia Japonica*.

[9] Oka H, Yamamoto A, Kuroki T (1995). Prospective study of chemoprevention of hepatocellular carcinoma with Shosaiko-to (TJ-9). *Cancer*.

[10] Qin X-K, Li P, Han M, Liu J-P (2010). Xiaochaihu Tang for treatment of chronic hepatitis B: a systematic review of randomized trials. *Journal of Chinese Integrative Medicine*.

[11] Deng G, Kurtz RC, Vickers A (2011). A single arm phase II study of a Far-Eastern traditional herbal formulation (sho-sai-ko-to or xiao-chai-hu-tang) in chronic hepatitis C patients. *Journal of Ethnopharmacology*.

[12] Shimizu I (2000). Sho-saiko-to: Japanese herbal medicine for protection against hepatic fibrosis and carcinoma. *Journal of Gastroenterology and Hepatology*.

[13] Lee J-K, Kim J-H, Shin HK (2011). Therapeutic effects of the oriental herbal medicine Sho-saiko-to on liver cirrhosis and carcinoma. *Hepatology Research*.

[14] Bo QL, Du WW (2006). Effect observation of fifty chronic hepatitis B patients under the treatment of interferon combined with xiaochaihu tang. *Shandong Medical Journal*.

[15] Li Z, Liao HH, Wu MJ, Lin ZH (2001). Study on the combination treatment of *γ*-interferon and xiaochaihu tang on liver fibrosis. *Chinese Journal of Integrated Traditional and Western Medicine on Liver Diseases*.

[16] Sun WH, Song MQ, Liu ZJ (2003). Study on the combination treatment of xiaochaihu tang and oxymatrine on hepatitis and hepato-fibrosis. *Chinese Journal of Integrated Traditional and Western Medicine on Liver Diseases*.

[17] Wu QQ (2009). Clinical observation on the effect of xiaochaihu tang on chronic hepatitis B with cirrhosis patients. *Journal of Zhejiang College of Traditional Chinese Medicine*.

[18] Yano H, Mizoguchi A, Fukuda K (1994). The herbal medicine sho-saiko-to inhibits proliferation of cancer cell lines by inducing apoptosis and arrest at the G0/G1 phase. *Cancer Research*.

[19] Makino T, Tsubouchi R, Murakami K, Haneda M, Yoshino M (2006). Generation of reactive oxygen species and induction of apoptosis of HL60 cells by ingredients of traditional herbal medicine, Sho-saiko-to. *Basic and Clinical Pharmacology and Toxicology*.

[20] Zhu K, Fukasawa I, Furuno M (2005). Inhibitory effects of herbal drugs on the growth of human ovarian cancer cell lines through the induction of apoptosis. *Gynecologic Oncology*.

[21] Li J, Xie M, Gan Y (2008). Effect of Xiaochaihu decoction and different herbal formulation of component on inhibiting H22 liver cancer in mice and enhancing immune function. *Zhongguo Zhongyao Zazhi*.

[22] Chang J-S, Wang K-C, Liu H-W, Chen M-C, Chiang L-C, Lin C-C (2007). Sho-saiko-to (Xiao-Chai-Hu-Tang) and crude saikosaponins inhibit Hepatitis B virus in a stable HBV-producing cell line. *American Journal of Chinese Medicine*.

[23] Cheng P-W, Ng L-T, Lin C-C (2006). Xiao Chai Hu Tang inhibits CVB1 virus infection of CCFS-1 cells through the induction of type I interferon expression. *International Immunopharmacology*.

[24] Cucchetti A, Cescon M, Trevisani F, Pinna AD (2012). Current concepts in hepatic resection for hepatocellular carcinoma in cirrhotic patients. *World Journal of Gastroenterology*.

[25] Kusunose M, Qiu B, Cui T (2002). Effect of Sho-saiko-to extract on hepatic inflammation and fibrosis in dimethylnitrosamine induced liver injury rats. *Biological and Pharmaceutical Bulletin*.

[26] Chen M-H, Chen J-C, Tsai C-C (2005). The role of TGF-β1 and cytokines in the modulation of liver fibrosis by Sho-saiko-to in rat’s bile duct ligated model. *Journal of Ethnopharmacology*.

[27] Dun BS (2011). *Fang Ji Xue*.

[28] Lee B, Shim I, Lee H, Hahm D-H (2009). Effect of Bupleurum falcatum on the stress-induced impairment of spatial working memory in rats. *Biological and Pharmaceutical Bulletin*.

[29] Yen M-H, Weng T-C, Liu S-Y, Chai C-Y, Lin C-C (2005). The hepatoprotective effect of Bupleurum kaoi, an endemic plant to Taiwan, against dimethylnitrosamine-induced hepatic fibrosis in rats. *Biological and Pharmaceutical Bulletin*.

[30] Kim SM, Kim SC, Chung IK, Cheon WH, Ku SK (2012). Antioxidant and protective effects of Bupleurum falcatum on the L-thyroxine-induced hyperthyroidism in rats. *Evidence-Based Complementary and Alternative Medicine*.

[31] Wu S-J, Tam K-W, Tsai Y-H, Chang C-C, Chao JC-J (2010). Curcumin and saikosaponin a inhibit chemical-induced liver inflammation and fibrosis in rats. *American Journal of Chinese Medicine*.

[32] Dang S-S, Wang B-F, Cheng Y-A, Song P, Liu Z-G, Li Z-F (2007). Inhibitory effects of saikosaponin-d on CCl_4_-induced hepatic fibrogenesis in rats. *World Journal of Gastroenterology*.

[33] Wu W-S, Hsu H-Y (2001). Involvement of p-15^INK4b^ and p-16^INK4a^ gene expression in saikosaponin a and TPA-induced growth inhibition of HepG2 cells. *Biochemical and Biophysical Research Communications*.

[34] Wu W-S (2003). ERK signaling pathway is involved in p15^INK4b^/p16^INK4a^ expression and HepG2 growth inhibition triggered by TPA and Saikosaponin a. *Oncogene*.

[35] Lu X-L, He S-X, Ren M-D, Wang Y-L, Zhang Y-X, Liu E-Q (2012). Chemopreventive effect of saikosaponin-d on diethylinitrosamine-induced hepatocarcinogenesis: involvement of CCAAT/enhancer binding protein β and cyclooxygenase-2. *Molecular Medicine Reports*.

[36] Zhu BH, Pu R, Zhang GP, Li MY, Wang LT, Yuan JK (2011). Effect of Saikosaponins-d on reversing malignant phenotype of HepG2 cells in vitro. *Zhonghua Gan Zang Bing Za Zhi*.

[37] Chiang L-C, Ng LT, Liu L-T, Shieh D-E, Lin C-C (2003). Cytotoxicity and anti-hepatitis B virus activities of saikosaponins from Bupleurum species. *Planta Medica*.

[38] Wang Q, Zheng X-L, Yang L (2010). Reactive oxygen species-mediated apoptosis contributes to chemosensitization effect of saikosaponins on cisplatin-induced cytotoxicity in cancer cells. *Journal of Experimental and Clinical Cancer Research*.

[39] Li-Weber M (2009). New therapeutic aspects of flavones: the anticancer properties of Scutellaria and its main active constituents Wogonin, Baicalein and Baicalin. *Cancer Treatment Reviews*.

[40] Ikemoto S, Sugimura K, Yoshida N (2000). Antitumor effects of Scutellariae radix and its components baicalein, baicalin, and wogonin on bladder cancer cell lines. *Urology*.

[41] Zhang DY, Wu J, Ye F (2003). Inhibition of cancer cell proliferation and prostaglandin E2 synthesis by Scutellaria baicalensis. *Cancer Research*.

[42] Gao J, Morgan WA, Sanchez-Medina A, Corcoran O (2011). The ethanol extract of Scutellaria baicalensis and the active compounds induce cell cycle arrest and apoptosis including upregulation of p53 and Bax in human lung cancer cells. *Toxicology and Applied Pharmacology*.

[43] Jung H-S, Kim MH, Gwak N-G (2012). Antiallergic effects of Scutellaria baicalensis on inflammation in vivo and in vitro. *Journal of Ethnopharmacology*.

[44] Tang Z-M, Peng M, Zhan C-J (2003). Screening 20 Chinese herbs often used for clearing heat and dissipating toxin with nude mice model of hepatitis C viral infection. *Zhongguo Zhong Xi Yi Jie He Za Zhi*.

[45] Tseng YP, Wu YC, Leu YL, Yeh SF, Chou CK (2010). Scutellariae radix suppresses hepatitis B virus production in human hepatoma cells. *Frontiers in Bioscience*.

[46] Wiley SR, Schooley K, Smolak PJ (1995). Identification and characterization of a new member of the TNF family that induces apoptosis. *Immunity*.

[47] James BR, Griffith TS (2012). Activation of systemic antitumor immunity via TRAIL-induced apoptosis. *Oncoimmunology*.

[48] Ding J, Polier G, Köhler R, Giaisi M, Krammer PH, Li-Weber M (2012). Wogonin and related natural flavones overcome tumor necrosis factor-related apoptosis-inducing ligand (TRAIL) protein resistance of tumors by down-regulation of c-FLIP protein and up-regulation of TRAIL receptor 2 expression. *The Journal of Biological Chemistry*.

[49] Polier G, Ding J, Konkimalla BV (2011). Wogonin and related natural flavones are inhibitors of CDK9 that induce apoptosis in cancer cells by transcriptional suppression of Mcl-1. *Cell Death & Disease*.

[50] Wang W, Guo Q, You Q (2006). Involvement of bax/bcl-2 in wogonin-induced apoptosis of human hepatoma cell line SMMC-7721. *Anti-Cancer Drugs*.

[51] Lin C-C, Kuo C-L, Lee M-H (2011). Wogonin triggers apoptosis in human osteosarcoma U-2 OS cells through the endoplasmic reticulum stress, mitochondrial dysfunction and caspase-3-dependent signaling pathways. *International Journal of Oncology*.

[52] Guo Q, Zhao L, You Q (2007). Anti-hepatitis B virus activity of wogonin in vitro and in vivo. *Antiviral Research*.

[53] Liang RR, Zhang S, Qi JA (2012). Preferential inhibition of hepatocellular carcinoma by the flavonoid Baicalein through blocking MEK-ERK signaling. *International Journal of Oncology*.

[54] Kuo H-M, Tsai H-C, Lin Y-L (2009). Mitochondrial-dependent caspase activation pathway is involved in baicalein-induced apoptosis in human hepatoma J5 cells. *International Journal of Oncology*.

[55] Chiu Y-W, Lin T-H, Huang W-S (2011). Baicalein inhibits the migration and invasive properties of human hepatoma cells. *Toxicology and Applied Pharmacology*.

[56] Sun H, Che Q-M, Zhao X, Pu X-P (2010). Antifibrotic effects of chronic baicalein administration in a CCl_4_ liver fibrosis model in rats. *European Journal of Pharmacology*.

[57] Zhang X, Tang X, Liu H, Li L, Hou Q, Gao J (2012). Autophagy induced by baicalin involves downregulation of CD147 in SMMC-7721 cells in vitro. *Oncology Reports*.

[58] Qiao H, Tong Y, Han H (2011). A novel therapeutic regimen for hepatic fibrosis using the combination of mesenchymal stem cells and baicalin. *Pharmazie*.

[59] Cheng Y, Ping J, Xu H-D, Fu H-J, Zhou Z-H (2006). Synergistic effect of a noval oxymatrine-baicalin combination against hepatitis B virus replication, *α* smooth muscle actin expression and type I collagen synthesis in vitro. *World Journal of Gastroenterology*.

[60] Qiao H, Han H, Hong D, Ren Z, Chen Y, Zhou C (2011). Protective effects of baicalin on carbon tetrachloride induced liver injury by activating PPAR*γ* and inhibiting TGFβ1. *Pharmaceutical Biology*.

[61] Park S-W, Lee C-H, Kim YS (2008). Protective effect of baicalin against carbon tetrachloride-induced acute hepatic injury in mice. *Journal of Pharmacological Sciences*.

[62] Kim S-J, Lee S-M (2012). Effect of baicalin on toll-like receptor 4-mediated ischemia/reperfusion inflammatory responses in alcoholic fatty liver condition. *Toxicology and Applied Pharmacology*.

[63] Jia L, Zhao Y, Liang X-J (2009). Current evaluation of the millennium phytomedicine—ginseng (II): collected chemical entities, modern pharmacology, and clinical applications emanated from traditional chinese medicine. *Current Medicinal Chemistry*.

[64] Helms S (2004). Cancer prevention and therapeutics: Panax ginseng. *Alternative Medicine Review*.

[65] Yun TK, Choi SY, Yun HY (2001). Epidemiological study on cancer prevention by ginseng: are all kinds of cancers preventable by ginseng?. *Journal of Korean Medical Science*.

[66] Wu XG, Zhu DH, Li X (2001). Anticarcinogenic effect of red ginseng on the development of liver cancer induced by diethylnitrosamine in rats. *Journal of Korean Medical Science*.

[67] Kwon Y-S, Jang K-H, Jang I-H (2003). The effects of Korean red ginseng (ginseng radix rubra) on liver regeneration after partial hepatectomy in dogs. *Journal of Veterinary Science*.

[68] Bak M-J, Jun M, Jeong W-S (2012). Antioxidant and hepatoprotective effects of the red ginseng essential oil in H_2_O_2_-treated HepG2 cells and CCl_4_-treated mice. *International Journal of Molecular Sciences*.

[69] Lee JY, Jung KH, Morgan MJ (2012). Sensitization of TRAIL-induced cell death by 20S-Ginsenoside Rg3 via CHOP-mediated DR5 upregulation in human hepatocellular carcinoma cells. *Molecular Cancer Therapeutics*.

[70] Zhang C, Liu L, Yu Y, Chen B, Tang C, Li X (2012). Antitumor effects of ginsenoside Rg3 on human hepatocellular carcinoma cells. *Molecular Medicine Reports*.

[71] Jiang J-W, Chen X-M, Chen X-H, Zheng S-S (2011). Ginsenoside Rg3 inhibit hepatocellular carcinoma growth via intrinsic apoptotic pathway. *World Journal of Gastroenterology*.

[72] Park HM, Kim SJ, Kim JS, Kang HS (2012). Reactive oxygen species mediated ginsenoside Rg3-and Rh2-induced apoptosis in hepatoma cells through mitochondrial signaling pathways. *Food and Chemical Toxicology*.

[73] Yoon J-H, Choi Y-J, Lee S-G (2012). Ginsenoside Rh1 suppresses matrix metalloproteinase-1 expression through inhibition of activator protein-1 and mitogen-activated protein kinase signaling pathway in human hepatocellular carcinoma cells. *European Journal of Pharmacology*.

[74] Yoon J-H, Choi Y-J, Cha S-W, Lee S-G (2012). Anti-metastatic effects of ginsenoside Rd via inactivation of MAPK signaling and induction of focal adhesion formation. *Phytomedicine*.

[75] Lo Y-T, Tsai Y-H, Wu S-J, Chen J-R, Chao JC-J (2011). Ginsenoside Rb1 inhibits cell activation and liver fibrosis in rat hepatic stellate cells. *Journal of Medicinal Food*.

[76] Geng J, Peng W, Huang Y, Fan H, Li S (2010). Ginsenoside-Rg1 from Panax notoginseng prevents hepatic fibrosis induced by thioacetamide in rats. *European Journal of Pharmacology*.

[77] Chen CF, Lee JF, Wang D, Shen CY, Shen KL, Lin MH (2010). Water extract of Zizyphus Jujube attenuates ischemia/reperfusion-induced liver injury in rats (PP106). *Transplantation Proceedings*.

[78] Shen X, Tang Y, Yang R, Yu L, Fang T, Duan J-A (2009). The protective effect of Zizyphus jujube fruit on carbon tetrachloride-induced hepatic injury in mice by anti-oxidative activities. *Journal of Ethnopharmacology*.

[79] Habib SHM, Makpol S, Hamid NAA, Das S, Ngah WZW, Yusof YAM (2008). Ginger extract (Zingiber officinale) has anti-cancer and anti-inflammatory effects on ethionine-induced hepatoma rats. *Clinics*.

[80] Yusof YAM, Ahmad N, Das S, Sulaiman S, Murad NA (2008). Chemopreventive efficacy of ginger (Zingiber officinale) in ethionine induced rat hepatocarcinogenesis. *African Journal of Traditional, Complementary and Alternative Medicines*.

[81] Hu R, Zhou P, Peng YB (2012). 6-Shogaol induces apoptosis in human hepatocellular carcinoma cells and exhibits anti-tumor activity in vivo through endoplasmic reticulum stress. *Plos One*.

[82] Weng CJ, Chou CP, Ho CT, Yen GC (2012). Molecular mechanism inhibiting human hepatocarcinoma cell invasion by 6-shogaol and 6-gingerol. *Molecular Nutrition and Food Research*.

[83] Sabina EP, Pragasam SJ, Kumar S, Rasool M (2011). 6-gingerol, an active ingredient of ginger, protects acetaminophen-induced hepatotoxicity in mice. *Journal of Chinese Integrative Medicine*.

[84] Huo HZ, Wang B, Liang YK, Bao YY, Gu Y (2011). Hepatoprotective and antioxidant effects of licorice extract against CCl_4_-induced oxidative damage in rats. *International Journal of Molecular Sciences*.

[85] Sharma A, Rathore HS (2011). Prevention of acetaminophen induced hepatorenal damage in mice with rhizomes of Glycyrrhiza glabra. A histophysiological study. *Ancient Science of Life*.

[86] Gumpricht E, Dahl R, Devereaux MW, Sokol RJ (2005). Licorice compounds glycyrrhizin and 18β-glycyrrhetinic acid are potent modulators of bile acid-induced cytotoxicity in rat hepatocytes. *The Journal of Biological Chemistry*.

[87] Lee C-H, Park S-W, Kim YS (2007). Protective mechanism of glycyrrhizin on acute liver injury induced by carbon tetrachloride in mice. *Biological and Pharmaceutical Bulletin*.

[88] Folkman J (2007). Angiogenesis: an organizing principle for drug discovery?. *Nature Reviews Drug Discovery*.

[89] Yue PYK, Wong DYL, Wu PK (2006). The angiosuppressive effects of 20(R)- ginsenoside Rg3. *Biochemical Pharmacology*.

[90] Yue PYK, Wong DYL, Ha WY (2005). Elucidation of the mechanisms underlying the angiogenic effects of ginsenoside Rg1 in vivo and in vitro. *Angiogenesis*.

[91] Zhang K, Lu J, Mori T (2011). Baicalin increases VEGF expression and angiogenesis by activating the ERR*α*/PGC-1*α* pathway. *Cardiovascular Research*.

[92] Sagar SM, Yance D, Wong RK (2006). Natural health products that inhibit angiogenesis: a potential source for investigational new agents to treat cancer—part 2. *Current Oncology*.

